# Effects of combined application of pig manure composts prepared using different fermentation methods with chemical fertilizer on winter wheat yield, light-thermal physiology, and soil biological characteristics

**DOI:** 10.3389/fpls.2025.1711898

**Published:** 2025-10-31

**Authors:** Mingteng Wang, Jiaming Cai, Chong Zeng, Meng Li, Yilun Wang, Sainan Geng, Gang Li, Lantao Li

**Affiliations:** ^1^ College of Resources and Environment, Henan Agricultural University, Zhengzhou, China; ^2^ College of Mechanical & Electrical Engineering, Henan Agricultural University, Zhengzhou, China

**Keywords:** winter wheat, fermentation method, integrated organic-inorganic fertilization, plant growth and development, soil fertility, microbial community structure

## Abstract

**Purpose:**

This study examined the effects and underlying mechanisms of combining pig manure composts, produced by different fermentation methods, with chemical fertilizers on winter wheat yield, nutrient uptake, light–thermal physiology, soil fertility, and microbial communities.

**Methods:**

Field experiment was conducted in Yuanyang County, Henan Province (2023–2024) with the following treatments: no fertilizer (CK), chemical fertilizer (TK), combined organic–inorganic applications with 25:75 and 50:50 ratios. The 25:75 treatments included natural compost (TA1), water-controlled trough compost (TA2), acid-controlled trough compost (TA3), and trough compost (TA4) combined with chemical fertilizer; the 50:50 treatments included the same four compost types (TB1–TB4). Their effects on yield, nutrient uptake dynamics, and light–thermal traits were evaluated, alongside changes in soil physicochemical properties, available nitrogen distribution, aggregate structure, enzyme activities, and microbial community composition and diversity.

**Results:**

The compost-to-fertilizer ratio of 25:75 significantly increased yields, with TA3 performing best—42.26%, 6.43% and 12.68% higher than the CK, TK, and the average of other organic fertilizer treatments, respectively. TA3 also recorded the highest total nitrogen, phosphorus, and potassium uptake and the greatest average uptake rate. It markedly enhanced photosynthetic performance at all growth stages. Compost–fertilizer combinations improved soil nutrient levels and increased the proportion of large aggregates (≥0.25 mm). High-throughput sequencing revealed that acid-regulated compost altered microbial community structure and promoted the expansion of rare taxa. Moreover, partial least squares path model indicated that acid-regulated trough compost increased yield by elevating soil nutrient levels and improving wheat physiological status.

**Conclusion:**

25:75 acid-regulated compost–fertilizer combination significantly improved winter wheat yield, nutrient efficiency, light–thermal use, soil fertility, structure, and microbial diversity, providing a practical basis for efficient organic fertilizer use in sandy fluvo-aquic soils of China’s winter wheat regions.

## 1 Introduction

Wheat (*Triticum aestivum* L.) is one of China’s most important staple crops, accounting for approximately 19.9% of the nation’s total grain cultivation area and 19.6% of total grain output (NBS, 2024). In recent years, advances in agricultural technology have improved both wheat yield and production efficiency. However, increasing crop productivity per unit area remains a major challenge in the context of accelerating climate change, population growth, and shrinking arable land resources (Yan et al., 2020). Chemical fertilizers play a vital role in enhancing crop yield and quality and remain indispensable in agricultural production. Nevertheless, widespread overuse and inefficient application methods have caused significant environmental issues, including soil degradation, increased greenhouse gas emissions, and water eutrophication (Xing et al., 2023). In 2023, China’s total fertilizer consumption reached 50.217 million tons, yet the seasonal utilization rates of nitrogen, phosphorus, and potassium fertilizers in wheat production were only 26–31%, 10–20%, and 30–40%, respectively (CAGDRS et al., 2024). Excessive and improper fertilizer use, along with low nutrient use efficiency, not only wastes resources but also exacerbates soil acidification, compaction, and organic matter loss. In some regions, soil organic matter content has fallen below the critical threshold of 1.5%, posing serious risks to sustainable agriculture (Zhao et al., 2023). Nutrient imbalance and low fertilizer use efficiency are often more pronounced in specific soil types, particularly in sandy fluvo-aquic soils (Yin et al., 2022). Therefore, the development of green and efficient nutrient management strategies that simultaneously improve crop productivity and soil health is essential for ensuring food security and enhancing soil fertility and quality.

Organic fertilizers are rich in organic matter, essential nutrients, and beneficial microorganisms. When applied appropriately, they can significantly improve soil structure, enhance nutrient cycling, increase soil enzyme activity, and boost soil fertility (Li et al., 2020). However, organic fertilizers also have limitations, such as low concentrations of key nutrients, slow nutrient release, and the need for large application volumes, making them insufficient on their own to meet crop nutrient demands during a single growing season (Zhu et al., 2024; Ren et al., 2025). Studies indicate that neither sole nor excessive application of organic fertilizer can meet current food security needs in most regions of China. Only through the integrated application of organic and chemical fertilizers, balancing quick nutrient availability with long-term fertility, can the benefits of both be fully realized—simultaneously improving wheat yield and soil fertility (He et al., 2022; Dang and Wang, 2025). Research has shown that combined application of both organic and inorganic fertilizers significantly increases crop yield, dry matter accumulation, and nutrient uptake (Lu et al., 2024), boosts grain crude protein and amino acid content (Sun et al., 2022), improves canopy structure and photosynthetic rates (Liang et al., 2022), and increases fertilizer use efficiency under the same application rate (Ren et al., 2022). Moreover, combined fertilization is a practical approach to improving soil fertility and structure. It can significantly increase soil nutrient content, optimize nutrient ratios (He et al., 2022), and enhance humus content and enzyme activities (Li et al., 2023a; Yin et al., 2024; Ren et al., 2025). This approach also helps regulate the balance between soil NH_4_^+^-N and NO_3_^-^-N dynamics and reduces NH_3_ volatilization losses (Watts et al., 2010; Duan et al., 2024a). Organic fertilizers supply various nutrients that serve as rich carbon and nitrogen sources and energy for microorganisms. Proper application promotes a more balanced soil microbial community (Jin et al., 2022; Yu et al., 2024), facilitates the colonization and expression of functional microbes (e.g., nitrogen-fixers and phosphate-solubilizers), and inhibits the proliferation of soil-borne pathogens (Wu et al., 2022). Enhancement of microbial community structure also strengthens the soil’s carbon and nitrogen metabolic networks, providing a sustained biological driver for nutrient efficiency and healthy crop root development (Yin et al., 2022).

With the rapid expansion of livestock and poultry production and increasing intensification of farming, China generates approximately 3.8 billion tons of livestock and poultry manure annually. The comprehensive utilization rate of this waste rose from less than 60% in 2015 to 75% in 2020. Converting livestock waste into organic fertilizer is considered one of the most effective strategies for recycling agricultural waste resources (Wei et al., 2021; Zhang et al., 2023a). While fresh manure is rich in nutrients and organic matter, it also contains significant quantities of pathogens, heavy metals, and emerging contaminants like microplastics and antibiotic resistance genes. Direct application of untreated manure to farmland can lead to the transfer of these pollutants through microbial activity into crops and potentially throughout the food chain, posing risks to ecosystem safety and human health (Li et al., 2024a; Cheng et al., 2022; Braun et al., 2021). Therefore, the development of appropriate fermentation and composting methods for livestock manure is of both theoretical and practical significance for improving the safety, reliability, and value-added utilization of organic fertilizers. Accordingly, this study focuses on how different fermentation methods affect compost quality to enhance both its safety and agronomic efficiency, providing a novel perspective for optimizing manure recycling and utilization.

Aerobic composting converts livestock and poultry manure into organic fertilizer rich in humus and nutrients, offering a resource-efficient and safe method for manure management and utilization (Qiu et al., 2020). Trough composting, a mechanized aerobic fermentation system built on trough reactors, maintains dynamic oxygen levels through mechanical turning and forced aeration. This method ensures a high degree of homogenization and efficient degradation of volatile organic compounds, making it the mainstream manure treatment process for large-scale livestock farms (Qiu et al., 2020; Tweib et al., 2014). However, the dual stress of sustained high temperature and alkaline conditions during composting can inhibit the activity of core functional microbes such as thermophilic actinomycetes (Zhang et al., 2023b; Li et al., 2023b). These conditions also promote the conversion of sulfides into toxic intermediates like thiosulfates, impeding lignocellulose degradation and slowing humification. As a result, the final compost may show a low germination index and residual levels of phenolic compounds and free ammonia, indicating incomplete maturation and reduced safety and agronomic efficiency (Xu et al., 2024; Duan et al., 2024b). Therefore, in addition to temperature regulation, adjusting the pH of compost products is critical for ensuring compost quality. pH is a key factor in aerobic composting; excessively high pH levels increase ammonia volatilization, suppress microbial activity, reduce organic matter decomposition, and compromise compost effectiveness. For example, Wang et al. (2018) found that adding a mixture of zeolite and biochar to pig manure compost significantly reduced NH_3_ emissions. Yuan et al. (2018) reported that adding superphosphate reduced NH_3_ release and improved nutrient retention in the final product. Current research on compost maturation mainly focuses on parameter optimization and physical amendments, but these methods can be complex and rarely verify post-compost performance in crop systems. To address this issue, this study employed readily available acetic acid as a chemical regulator to control pH levels during the composting process and validated its impact on crop system performance through field experiment. A field experiment was then conducted using winter wheat, combining organic fertilizers produced by different fermentation methods—natural composting, water-regulated trough composting, acid-regulated trough composting, and conventional trough composting—with chemical fertilizers at two mixing ratios: 25:75 and 50:50 (organic: inorganic). This study aims to systematically investigate how pig manure compost produced by different fermentation methods, when combined with chemical fertilizers, affects winter wheat yield, light-thermal physiological traits, nutrient uptake and accumulation dynamics, and soil properties, including nutrient content, enzyme activity, aggregate structure, and microbial community composition. The findings are expected to provide technical guidance for the rational use of organic fertilizer, as well as for enhancing wheat yield and quality and improving cultivated land health through sustainable practices.

## 2 Materials and methods

### 2.1 Experimental site description

**Figure 1:**
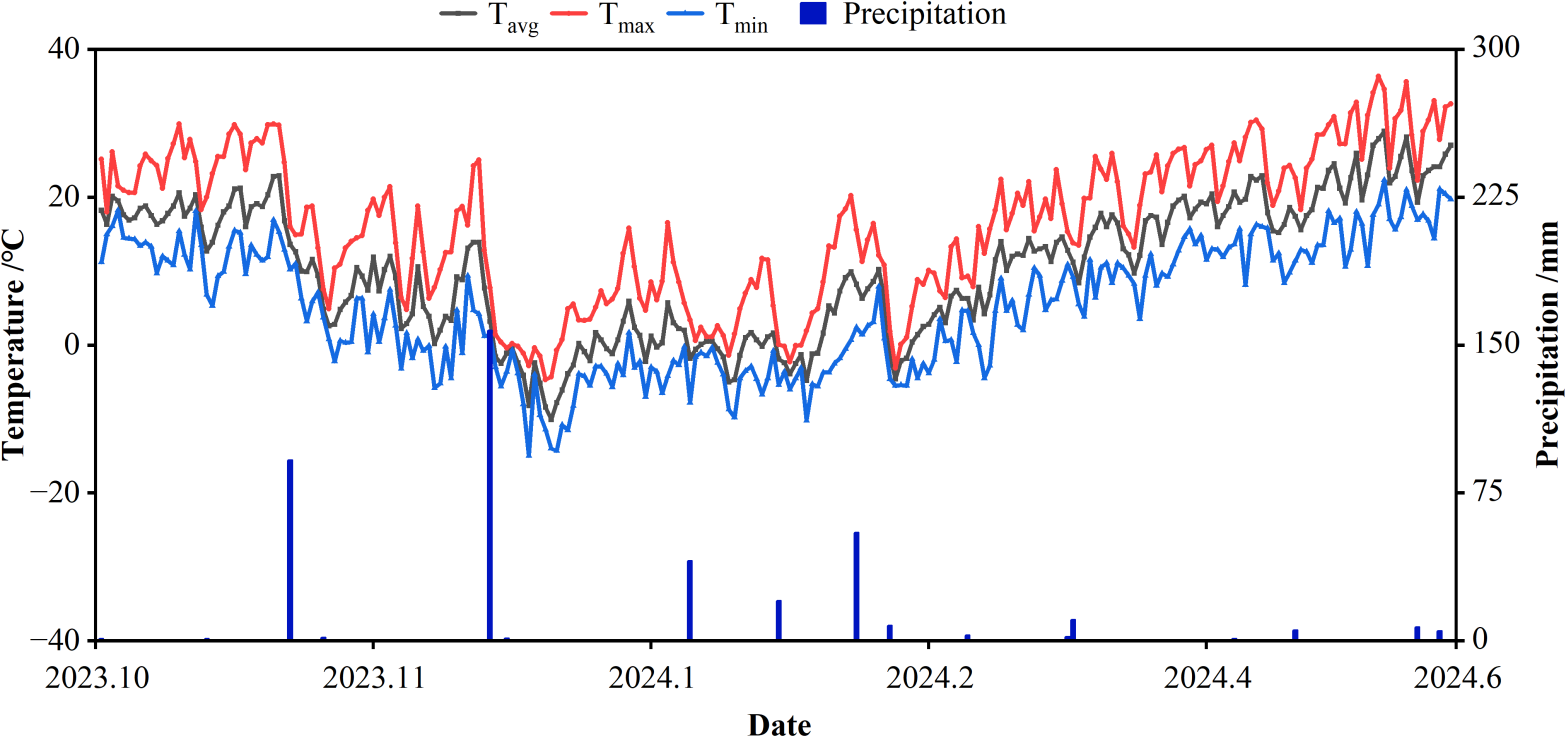
The temperature and rainfall of the test site during 2023–2024 growing season.

From October 2023 to June 2024, a field experiment was conducted at the Yuanyang Science and Education Park of Henan Agricultural University (35°11′N, 113°95′E), testing the effects of different fermentation methods of organic fertilizer combined with chemical fertilizer on winter wheat. Detailed temperature and precipitation trends during the experiment are shown in **Figure 1**. The test soil was sandy fluvo-aquic soil, with the 0–20 cm topsoil layer having the following baseline properties: pH 8.08, bulk density 1.36 g·cm^-^³, organic matter 16.17 g·kg^-^¹, total nitrogen (TN) 0.92 g·kg^-^¹, available phosphorus (AP) 18.41 mg·kg^-^¹, and available potassium (AK) 112 mg·kg^-^¹.

### 2.2 Experimental design

The organic fertilizers used in this study were prepared by fermenting fresh pig manure (after solid-liquid separation) with wheat straw for 32 days. Four fermentation methods were applied. The first was natural composting, in which the pig manure and straw were stacked in windrows measuring 10 m in length, 2.5 m in width, and 1.2 m in height for passive fermentation. The remaining three methods involved trough composting, conducted in a trough structure measuring 40 m in length (divided into three 10 m sections with 5 m buffer zones), 20 m in width, and 2.5 m in height. The compost was turned every 24 hours using mechanical equipment. The three trough composting treatments were as follows: (1) Conventional trough composting, where no additional treatment was applied before or after turning; (2) Water-Regulated trough composting method: After each turning, spray water onto the compost surface using a sprayer at a rate of 2 kg per square meter. (3) Acid-regulated trough composting method: After each turning, spray a 0.005 mol·L^-^¹ dilute acetic acid solution onto the compost surface at a rate of 2 kg per square meter. The physicochemical properties of the organic fertilizers before and after fermentation are shown in **Table 1**.

**Table 1:** Physicochemical properties of the organic manure before fermentation and after fermentation.

Time	Organic matter	pH	Water content (%)	Total N (%)	Total P (%)	Total K (%)
Before fermentation	Fresh pig manure	7.92	75.36	0.98	1.00	1.88
	Straw	6.74	12.28	1.04	0.91	2.44
After fermentation	Natural composting	7.91	43.63	0.98	1.00	1.88
	Water-regulated trough composting	7.68	34.21	1.04	0.91	2.44
	Acid-regulated trough composting	7.53	34.13	1.16	1.14	2.71
	Trough composting	7.81	32.59	1.17	0.98	2.43

A total of 10 treatments were established in the field experiment: no fertilization (CK); chemical fertilizer only (TK); and two ratios of organic–inorganic fertilizer combinations. For the 25% organic fertilizer + 75% chemical fertilizer treatments: natural compost + fertilizer (TA1), water-regulated trough compost + fertilizer (TA2), acid-regulated trough compost + fertilizer (TA3), and conventional trough compost + fertilizer (TA4). For the 50% organic fertilizer + 50% chemical fertilizer treatments: natural compost + fertilizer (TB1), water-regulated trough compost + fertilizer (TB2), acid-regulated trough compost + fertilizer (TB3), and conventional trough compost + fertilizer (TB4).

Application rates for nitrogen (N), phosphorus (P_205_), and potassium (K_2_O) were 150, 120, and 90 kg·hm^-^², respectively. Organic fertilizers substituted for 25% or 50% of the total chemical fertilizer input based primarily on nitrogen content and adjusted for phosphorus and potassium. The chemical fertilizers used were urea (46% N), single superphosphate (12% P_205_), and potassium chloride (60% K_2_O), all applied as basal fertilizer once before sowing. Each plot measured 36 m² (3.6 m × 10 m), with three replicates arranged in a randomized block design. The winter wheat variety used was ‘Zhengmai 369’, sown on October 17, 2023, and harvested on May 29, 2024, at a seeding rate of 300 kg·hm^-^². Field management followed local farming practices.

### 2.3 Measurement items and methods

#### 2.3.1 Yield measurement

At wheat maturity, all grain spikes within a uniformly growing 5.0 m² area of each plot were harvested, air-dried under natural conditions, threshed, and weighed to calculate grain yield.

#### 2.3.2 Plant sample collection and analysis

At the overwintering, jointing, flowering, and maturity stage, representative 1 m double-row wheat samples were collected from each plot. The samples were inactivated at 105°C for 30 minutes, dried at 75°C to a constant weight, and then weighed to determine biomass. After grinding, samples were digested using the concentrated H_2_SO_4_-H_2_O_2_ method. TN was measured using the Kjeldahl method, total phosphorus by the molybdenum blue colorimetric method, and total potassium using flame photometry (Bao, 2000).

### 2.4 Canopy temperature measurement and analysis

At the overwintering, jointing, and flowering stage, canopy temperature was measured between 11:00 and 13:00 on sunny days with minimal wind using an infrared thermal imager (TiX650, Fluke, USA). The imager’s camera lens was positioned approximately 1.0 m above the canopy with a 60° viewing angle. Three images were captured per plot, and canopy temperature were analyzed using the instrument’s SmartView software.

### 2.5 Leaf photosynthetic parameter measurement

At the overwintering and jointing stage, the newest fully expanded leaves were selected (flag leaves at flowering). Between 9:00 and 11:00 AM, photosynthetic parameters, including net photosynthetic rate (Pn, µmol·m^-^²·s^-^¹), stomatal conductance (Gs, mol·m^-^²·s^-^¹), intercellular CO_2_ concentration (Ci, µmol·mol^-^¹), and transpiration rate (Tr, μmol·m^-^²·s^-^¹), were measured using the LI-6800 portable photosynthesis system (LI-COR, USA), with the leaf chamber setting to a light intensity of 1000 µmol·m^-^²·s^-^¹, CO_2_ concentration of 400 µmol·mol^-^¹, relative humidity of 50%, temperature of 25°C, and airflow rate of 500 µmol·s^-^¹ (Liang et al., 2022).

### 2.6 Soil sampling and analysis

For soil nutrient measurement, soil samples were collected from the 0–20 cm layer before sowing and fertilization and at the overwintering, jointing, flowering, and maturity stage. After air-drying and sieving, soil pH, organic matter, TN, AP and AK were tested (Bao, 2000). For soil aggregates and C: N ratio measurement, three soil samples were collected from the 0–20 cm layer per plot at the overwintering, jointing, flowering, and maturity stage. Samples from the same stage were mixed, fractionated using the wet-sieving method following Cambardella and Elliott (1993). Proportions of >2 mm (macro-aggregates), 0.25–2 mm (large aggregates), 0.053–0.25 mm (micro-aggregates), and <0.053 mm (silt and clay) were determined. TN and organic carbon were measured for each aggregate size class (Bao, 2000). For soil enzyme activity measurement, fresh soil samples were collected at the overwintering, jointing, and flowering stage. Urease activity (S-URE) was measured by the phenol-sodium hypochlorite colorimetric method, acid phosphatase (S-ACP) by the disodium phenyl phosphate method, and dehydrogenase activity (S-DHA) by potassium permanganate titration (Wang et al., 2020).

For soil microbial community composition analysis, fresh soil samples were collected at wheat maturity and stored in liquid nitrogen. Samples were ground in extraction buffer using a Tissuelyser-48 (Jingxin Instruments, Shanghai, China) at 60 Hz. DNA was extracted using the MagBeads FastDNA Kit for Soil (MP Biomedicals, USA) and quantified with a Nanodrop spectrophotometer. Bacterial 16S rRNA V3–V4 region and fungal ITS1 region were amplified using primer pairs 338F (5’-barcode+ACTCCTACGGGAGGCAGCA-3’)/806R (5’-GGACTACHVGGGTWTCTAAT-3’) and ITS5 (GGAAGTAAAAGTCGTAACAAGG)/ITS2 (GCTGCGTTCTTCATCGATGC), respectively (Gade et al., 2013) and sequenced.

### 2.7 Data analysis and processing

The nutrient accumulation process of winter wheat followed an S-shaped curve that conformed to the logistic model. A logistic equation was used to fit the dynamic accumulation of nitrogen (N), phosphorus (P), and potassium (K) under different treatments, and the corresponding parameters were calculated as follows (Yin et al., 2018):


(1) y=k/(1+exp^(a−b*x) )

where k, a, and b are constants; y is the nutrient accumulation (N, P, or K) at x days after sowing. By differentiating the equation, the following parameters were derived:


(2) t_1=(lna−1.317)/b

(3) t_2=(lna+1.317)/b

(4) $$\Delta_{t}=t_{2}-t_{1}$$

(5) T_max=lna/b

(6) V_max=k*b/4

(7) V_mean=k*b/6

where t_1_ is the starting time of the rapid accumulation phase, t_2_ is the end time, Δt is the duration of rapid nutrient accumulation, T_max_ is the time of maximum accumulation rate, V_max_ is the maximum accumulation rate, and V_mean_ is the average accumulation rate.

All basic data processing and analyses were performed using Microsoft Excel 2021. SPSS Statistics 22.0 was employed for analysis of variance (ANOVA) and significance testing with the LSD method. DPS 7.05 was used for Logistic equation fitting, and Origin Pro 2024 was used for plotting. Microbial data were statistically analyzed using R software (version 4.5.0), with packages linkET, ggplot2, ggtext, dplyr, RColorBrewer, cols4all, and tidy verse for Mantel tests. Partial least squares path model was used to explore the reasons for the increase of wheat yield by pig manure composting. The path coefficient and determination coefficient in the path model were measured and verified by plspm package. Canoco 5 for redundancy analysis (RDA).

## 3 Results

### 3.1 Yield and yield components

The combined application of pig manure compost prepared under different fermentation methods and chemical fertilizer significantly improved winter wheat yield and its yield components (**Table 2**). Compared with CK, all treatments (TK–TB4) increased spike grain number, thousand-grain weight, and grain yield by 6.33%–17.07%, 1.55%–7.14%, and 17.72%–49.27%, respectively. Among treatments with 25% organic fertilizer and 75% chemical fertilizer (TA1–TA4), yield components improved further compared to TK, with average increases of 2.11%–4.03% in spike grain number, 1.51%–3.88% in thousand-grain weight, and 0.93%–6.87% in yield. The TA3 treatment showed the best yield performance, with significant increases of 1.62 g in thousand grain weight and 525.71 kg·hm^-^² in grain yield compared to TK (*P* < 0.05). In contrast, treatments with 50% organic and 50% chemical fertilizer (TB1–TB4) resulted in lower yield than TK, with reductions in spike grain number, thousand grain weight, and yield ranging from 0.60%–5.51%, 0.22%–1.53%, and 9.84%–11.57%, respectively.

**Table 2:** Effects of different fermentation methods of pig manure compost combined with chemical fertilizer on winter wheat yield and its components.

Treatment	Grain number per spike	Thousand seed weight (g)	Yield (kg·hm^-2^)
CK	29.53 ± 1.16 b	40.46 ± 1.12 d	5749.92 ± 254.08 d
TK	33.23 ± 2.96 a	41.73 ± 0.78 bcd	7654.17 ± 339.81 b
TA1	34.03 ± 1.16 a	42.97 ± 0.20 ab	8015.60 ± 300.68 ab
TA2	34.30 ± 1.57 a	41.81 ± 0.55 bcd	7991.80 ± 200.11 ab
TA3	34.57 ± 1.37 a	43.35 ± 0.68 a	8179.88 ± 194.30 a
TA4	33.93 ± 2.03 a	42.36 ± 1.03 abc	7725.61 ± 345.79 ab
TB1	31.83 ± 1.32 ab	41.09 ± 0.64 cd	6848.45 ± 495.87 c
TB2	33.03 ± 1.69 a	41.45 ± 0.74 bcd	6834.19 ± 205.19 c
TB3	32.53 ± 1.30 ab	41.64 ± 1.25 bcd	6901.33 ± 98.60 c
TB4	31.40 ± 2.62 ab	41.55 ± 0.56 bcd	6768.47 ± 542.88 c

Different lowercase letters in the same row indicate significant differences between treatments (*P* < 0.05), the same below.

### 3.2 Above-ground nutrient accumulation dynamics

**Figure 2:**
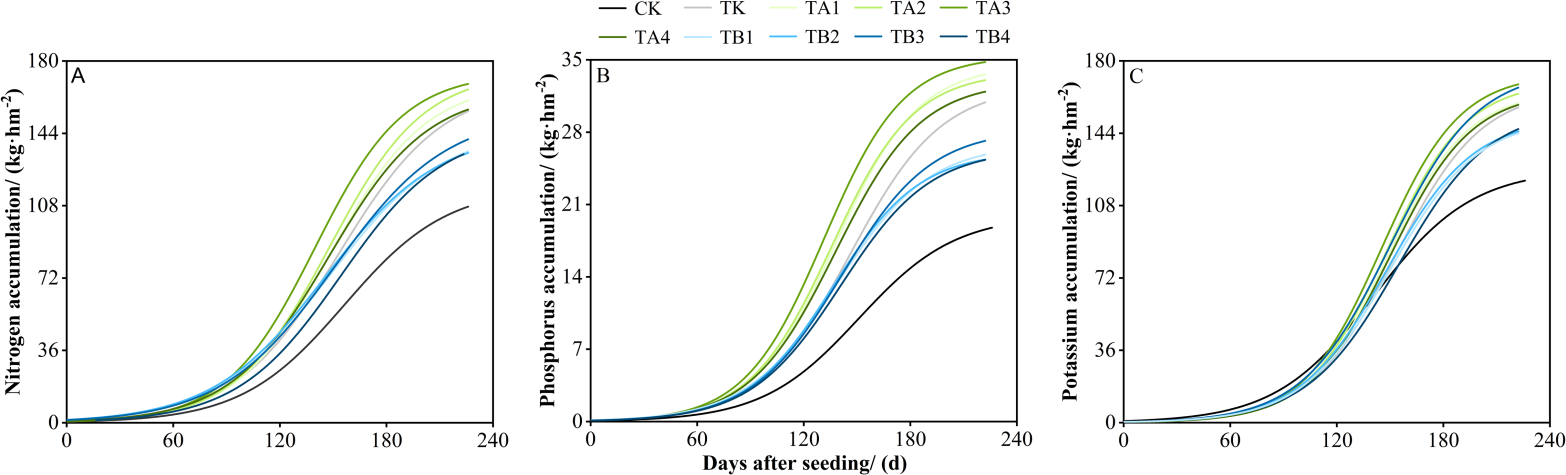
Effects of different fermentation methods of pig manure compost combined with chemical fertilizer on the dynamics of nutrient accumulation in the aboveground part of winter wheat.

Across all treatments, N, P, and K accumulation in the above-ground parts of winter wheat followed an S-shaped curve over the growing season, characterized by an initial slow increase, a subsequent rapid accumulation phase, and a final plateau (**Figure 2**). Model fitting (Equations 1–7) results (**Supplementary Table S1**) showed that compared to TK, all treatments with 25% pig manure compost and 75% chemical fertilizer (TA1–TA4) increased the maximum above-ground accumulation (Y_m_) of N, P, and K by 1.40–6.57, 0.37–2.88, and 3.86–7.60 kg·hm^-^², respectively. The TA3 treatment performed best, with Y_m_ values of 173.98, 35.38, and 173.60 kg·hm^-^², representing increases of 3.50%, 8.85%, and 4.58% over TK. TA2 ranked second, with respective increases of 3.90%, 4.03%, and 2.33%. In contrast, treatments with 50% organic fertilizer and 50% chemical fertilizer (TB1–TB4) significantly reduced above-ground N, P, and K accumulation, with reductions of 15.24–24.62, 6.25–6.40, and 9.38–14.35 kg·hm^-^², respectively. Furthermore, treatments TA1–TA4 advanced the onset of rapid nutrient accumulation (t_1_) by 1.0–7.6 days for N, 5.4–10.5 days for P, and 1.5–5.0 days for K, with TA3 showing the earliest onset. These treatments also shortened the duration of rapid accumulation (Δt) by 4.15–11.64 days (N), 4.59–9.60 days (P), and 3.46–5.78 days (K). Compared to TK, TA1–TA4 also enhanced average accumulation rates (V_mean_) for N, P, and K by 12.71%, 16.43%, and 9.11%, respectively. In summary, TA3 was the most effective treatment, characterized by the highest Y_m_, the greatest average accumulation rate (V_mean_), and the shortest duration of rapid accumulation (Δt).

### 3.3 Canopy temperature and photosynthetic parameters

**Figure 3:**
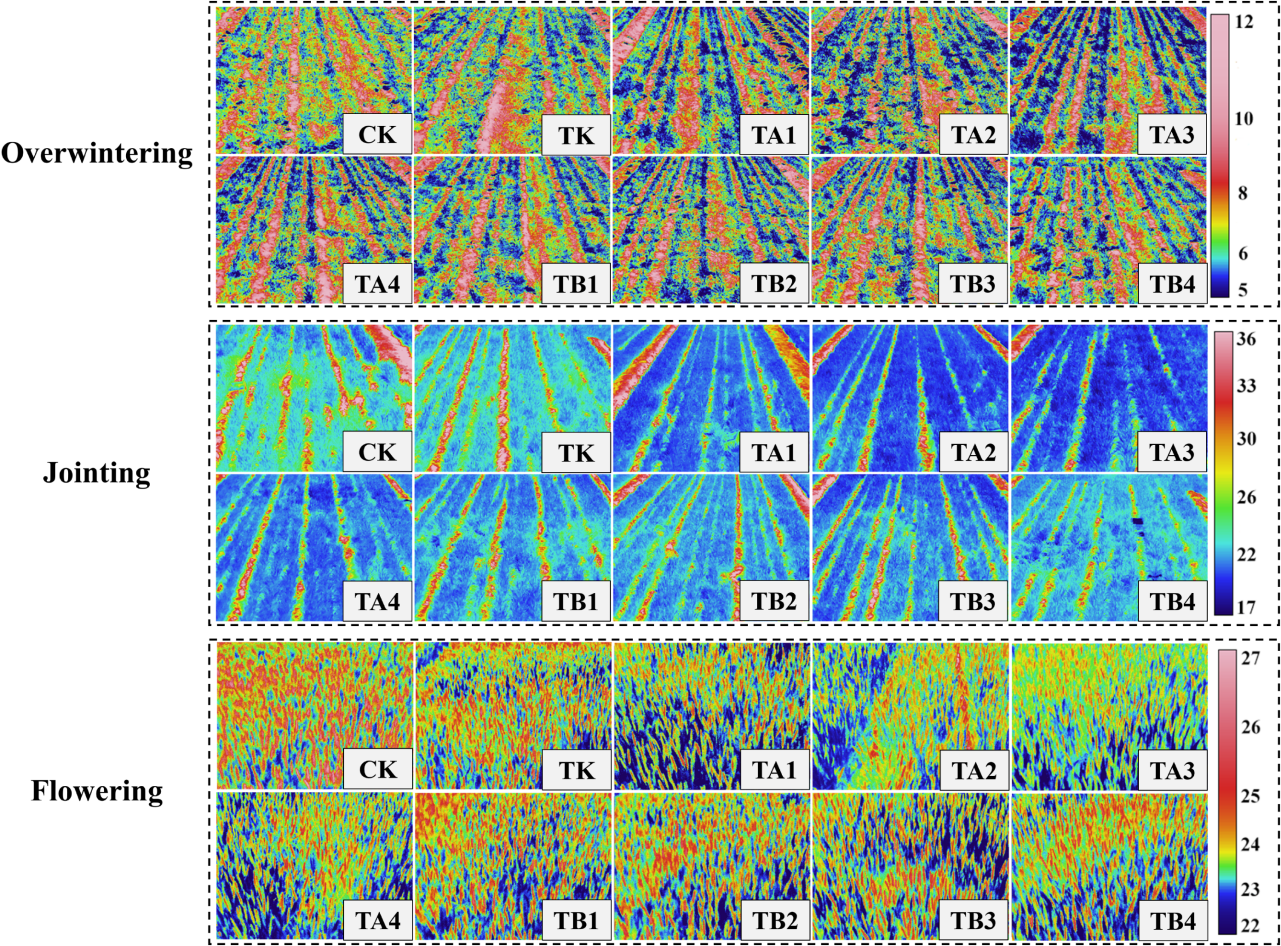
Effects of different fermentation methods of pig manure compost combined with chemical fertilizer on canopy temperature of winter wheat (thermal image, temperature scale in °C).

The application of pig manure compost under different fermentation methods with chemical fertilizers significantly affected canopy temperature and photosynthetic parameters of winter wheat (**Supplementary Table S2**, **Figure 3**). Compared to CK, all fertilizer treatments reduced the minimum, maximum, and average canopy temperature. During the overwintering, jointing, and flowering stage, the TK treatment reduced average canopy temperature by 0.13 °C, 0.51 °C, and 0.80 °C, respectively, compared to CK. Treatments with pig manure compost plus chemical fertilizer (TA1–TB4) further lowered canopy temperature, with average reductions of 7.04%, 2.36%, and 4.26% compared to TK. Among all treatments, TA3 performed best, with average canopy temperature of 6.93°C, 21.27°C, and 22.83°C during the overwintering, jointing, and flowering stage, respectively—5.33% lower than TK on average. In addition, co-application of pig manure compost and chemical fertilizer improved leaf photosynthetic performance more effectively than chemical fertilizer alone (**Supplementary Figure S1**). Pn, Gs, and Tr increased progressively over the growth stage, while Ci decreased. Compared with CK, the TK treatment significantly increased Pn, Gs, and Tr, while reducing Ci. Treatments TA1–TA4 further enhanced Pn, Gs, and Tr, with a greater decline in Ci. Conversely, treatments TB1–TB4 showed lower levels of Pn, Gs, and Tr than TK, and higher Ci levels, forming the trends of TA > TK > TB for Pn, Gs, and Tr and TA < TB < TK for Ci. Among all treatments, TA3 again performed best. At the flowering stage, Pn, Gs, and Tr peaked at 26.52 μmol·m^-^²·s^-^¹, 0.54 mol·m^-^²·s^-^¹, and 8.78 mmol·m^-^²·s^-^¹, respectively, while Ci reached its lowest value of 154.45 μmol·mol^-^¹.

### 3.4 Soil Enzyme activity and nutrient content

Soil enzyme activities increased progressively throughout the winter wheat growth stage and were significantly affected by fertilization (**Table 3**). The CK group exhibited the lowest activities of soil urease (S-URE), acid phosphatase (S-ACP), and dehydrogenase (S-DHA), with reductions of 34.04%, 81.11%, and 37.29%, respectively, compared to TK. Co-application of pig manure compost and chemical fertilizer significantly enhanced soil enzyme activity, with the TB1–TB4 treatments (50% organic fertilizer) showing the most pronounced effects. Compared to TK, TB1–TB4 S-URE activity by 15.82%, 20.74%, and 14.80%, S-ACP activity by 29.01%, 33.33%, and 55.36%, and S-DHA activity by 25.59%, 66.84%, and 14.50% during the overwintering, jointing, and flowering stage, respectively. Relative to the TA1–TA4 treatments (25% organic fertilizer), TB1–TB4 further improved soil enzyme activities (S-URE, S-ACP, and S-DHA) by 8.37%, 12.20%, and 15.12% on average during the overwintering, jointing, and flowering stage, respectively. Consistent with enzyme activity trends, soil nutrient levels were also significantly affected by fertilization (**Supplementary Figure S2**). At maturity, TK increased TN, AP, and AK by 28.41%, 91.54%, and 9.54%, respectively, over the CK group, while soil organic matter (SOM) rose by 2.61% (*P* > 0.05). On average, TA1–TB4 treatments raised TN, AP, AK, and SOM by 43.44%, 173.57%, 24.65%, and 19.04%, respectively. Compared to TK, TA1–TA4 increased TN, AP, AK, and SOM by 9.48%, 57.40%, 19.06%, and 9.18%, while TB1–TB4 increased these values by 13.94%, 28.26%, 8.53%, and 22.83%, respectively. In terms of pH, TK lowered soil pH by 0.14 compared to CK, whereas TA1–TB4 treatments reduced it by only 0.05 on average. Compared to TK, TA1–TA4 and TB1–TB4 increased soil pH by 0.10 and 0.08, respectively.

**Table 3:** Effects of different fermentation methods of pig manure compost and chemical fertilizer on soil enzyme activity of winter wheat (IU/L).

Treatment	Overwintering			Jointing			Flowering		
	S-URE	S-ACP	S-DHA	S-URE	S-ACP	S-DHA	S-URE	S-ACP	S-DHA
CK	854.31 ± 28.11c	21.54 ± 0.60e	32.91 ± 0.46g	877.76 ± 16.06g	23.62 ± 0.59g	41.39 ± 0.94f	866.36 ± 5.84f	22.53 ± 0.20g	42.64 ± 1.07g
TK	1066.50 ± 16.52e	37.27 ± 1.57d	48.98 ± 0.50f	1152.00 ± 11.81ef	41.23 ± 0.90f	46.74 ± 0.65e	1298.07 ± 28.79e	44.10 ± 0.76f	64.03 ± 1.13f
TA1	1188.37 ± 27.56bc	50.53 ± 0.60a	51.23 ± 1.70e	1239.76 ± 5.64c	45.07 ± 0.53e	48.73 ± 1.10de	1342.90 ± 37.19de	53.11 ± 1.00d	70.14 ± 0.63e
TA2	1140.16 ± 57.39cd	49.47 ± 1.08a	55.42 ± 1.01cd	1127.37 ± 15.74f	53.94 ± 1.08bc	65.78 ± 1.83c	1498.68 ± 37.52b	52.87 ± 0.76d	72.52 ± 1.71cd
TA3	1190.96 ± 43.84bc	40.21 ± 0.57c	56.78 ± 1.40c	1194.12 ± 14.14d	52.43 ± 0.43c	69.97 ± 0.85b	1444.37 ± 9.89c	61.08 ± 0.24c	70.75 ± 1.79de
TA4	1108.52 ± 34.02de	37.29 ± 0.49d	54.14 ± 1.07d	1175.22 ± 12.85de	48.67 ± 0.50d	52.15 ± 2.15de	1386.11 ± 46.20d	49.00 ± 0.73e	75.20 ± 0.61b
TB1	1112.91 ± 21.72de	49.90 ± 1.59a	59.25 ± 1.02b	1315.86 ± 25.65b	57.68 ± 2.43a	75.67 ± 3.06a	1338.66 ± 7.82de	61.76 ± 0.48c	71.43 ± 1.06cde
TB2	1264.32 ± 32.05a	43.43 ± 0.49b	62.82 ± 1.12a	1391.96 ± 42.23a	49.88 ± 0.48d	76.55 ± 2.20a	1500.90 ± 38.03b	70.04 ± 0.48a	73.48 ± 0.89bc
TB3	1292.86 ± 2.12a	49.80 ± 0.36a	60.07 ± 0.91b	1412.77 ± 26.68a	57.37 ± 0.32a	74.76 ± 3.88a	1576.93 ± 31.83a	68.23 ± 0.43b	79.05 ± 1.63a

### 3.5 Spatial distribution of NH_4_^+^-N and NO_3_^–^N

**Figure 4:**
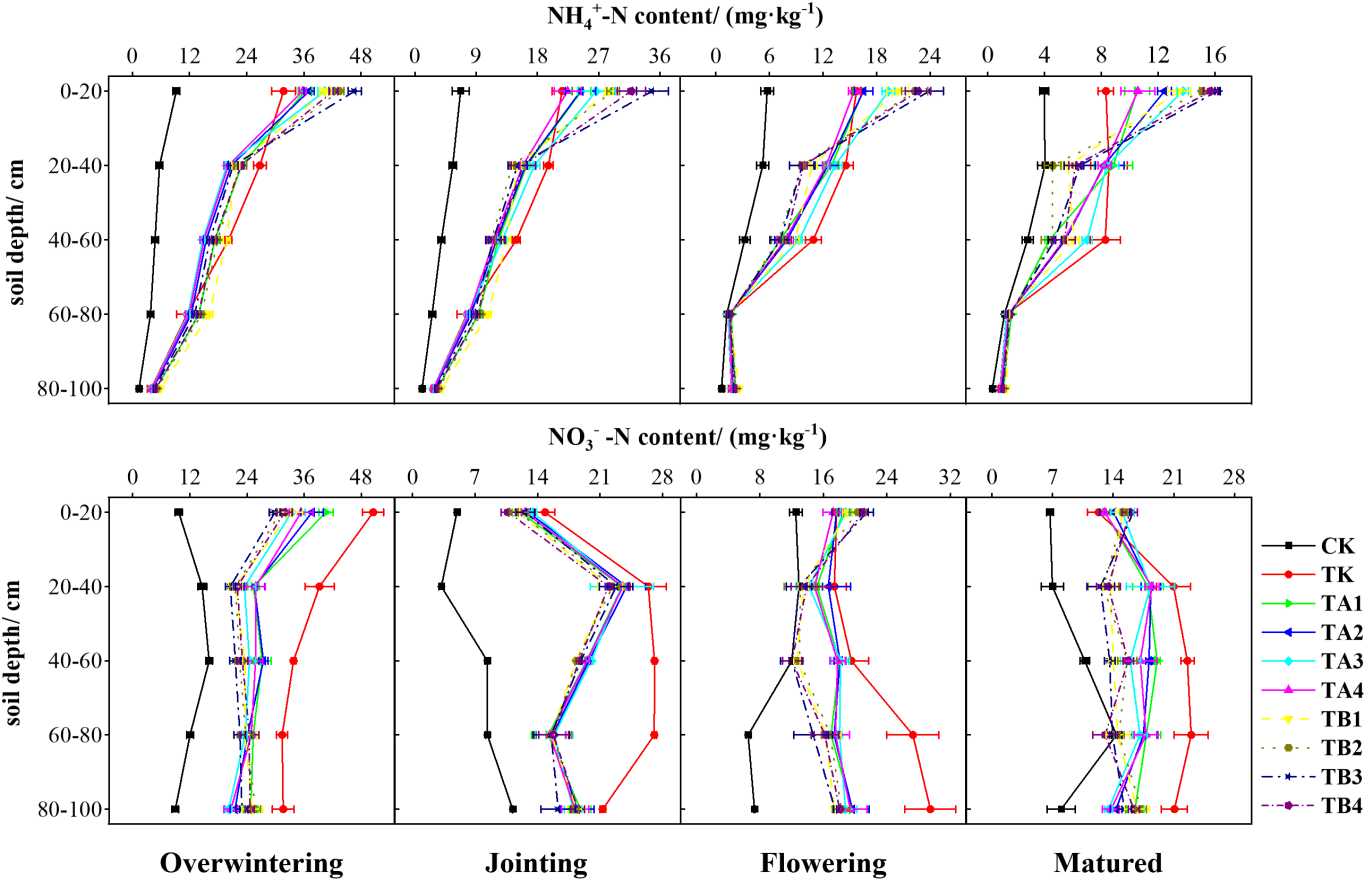
Effects of different fermentation methods of pig manure compost combined with chemical fertilizer on NH_4_^+^-N and NO_3_^-^-N contents in different soil layers of winter wheat.

The spatial distribution of soil NH_4_^+^-N and NO_3_^-^-N in the 0–100 cm profile was significantly affected by the combined application of pig manure compost and chemical fertilizer under different fermentation methods (**Figure 4**). TK treatment markedly increased NH_4_^+^-N and NO_3_^-^-N levels across all soil layers. In contrast, treatments TA1–TB4 enhanced NH_4_^+^-N concentrations primarily in the 0–20 cm layer while reducing NO_3_^-^-N accumulation in deeper layers (40–100 cm). During the overwintering stage, treatments TK–TB4 significantly increased NH_4_^+^-N in the 0–80 cm layer and NO_3_^-^-N in the 0–100 cm layer, both decreasing with soil depth. The most notable declines in NH_4_^+^-N and NO_3_^-^-N occurred between the 0–20 cm and 20–40 cm layers. At the jointing stage, NH_4_^+^-N maintained a depth-dependent decline similar to that observed during overwintering, whereas NO_3_^-^-N first increased and then decreased with depth. NO_3_^-^-N peaked at 60–80 cm (23.00 mg·kg^-^¹) in the TK group but peaked at 20–40 cm (12.88–18.00 mg·kg^-^¹) in the TA1–TB4 groups. During flowering, NH_4_^+^-N levels declined rapidly from 0–80 cm, with TA1–TB4 maintaining high concentrations in the top 0–20 cm layer (15.49–23.89 mg·kg^-^¹). At maturity, NH_4_^+^-N levels followed a pattern similar to that observed at flowering. In the 0–20 cm layer, NH_4_^+^-N concentrations under TA1–TB4 were 26.20%–94.40% higher than under TK. NO_3_^-^-N content under TK and TA1–TA4 initially increased with depth and then declined, with TK showing the highest NO_3_^-^-N accumulation in the 20–100 cm layer. In contrast, TB1–TB4 treatments showed an inverse trend—NO_3_^-^-N increased in the 0–20 cm layer by 7.84%–21.61% over TK, dropped to the lowest levels at 40–60 cm (12.03–13.00 mg·kg^-^¹), and then rose again in deeper layers.

### 3.6 Aggregate distribution and carbon-nitrogen ratio

**Figure 5:**
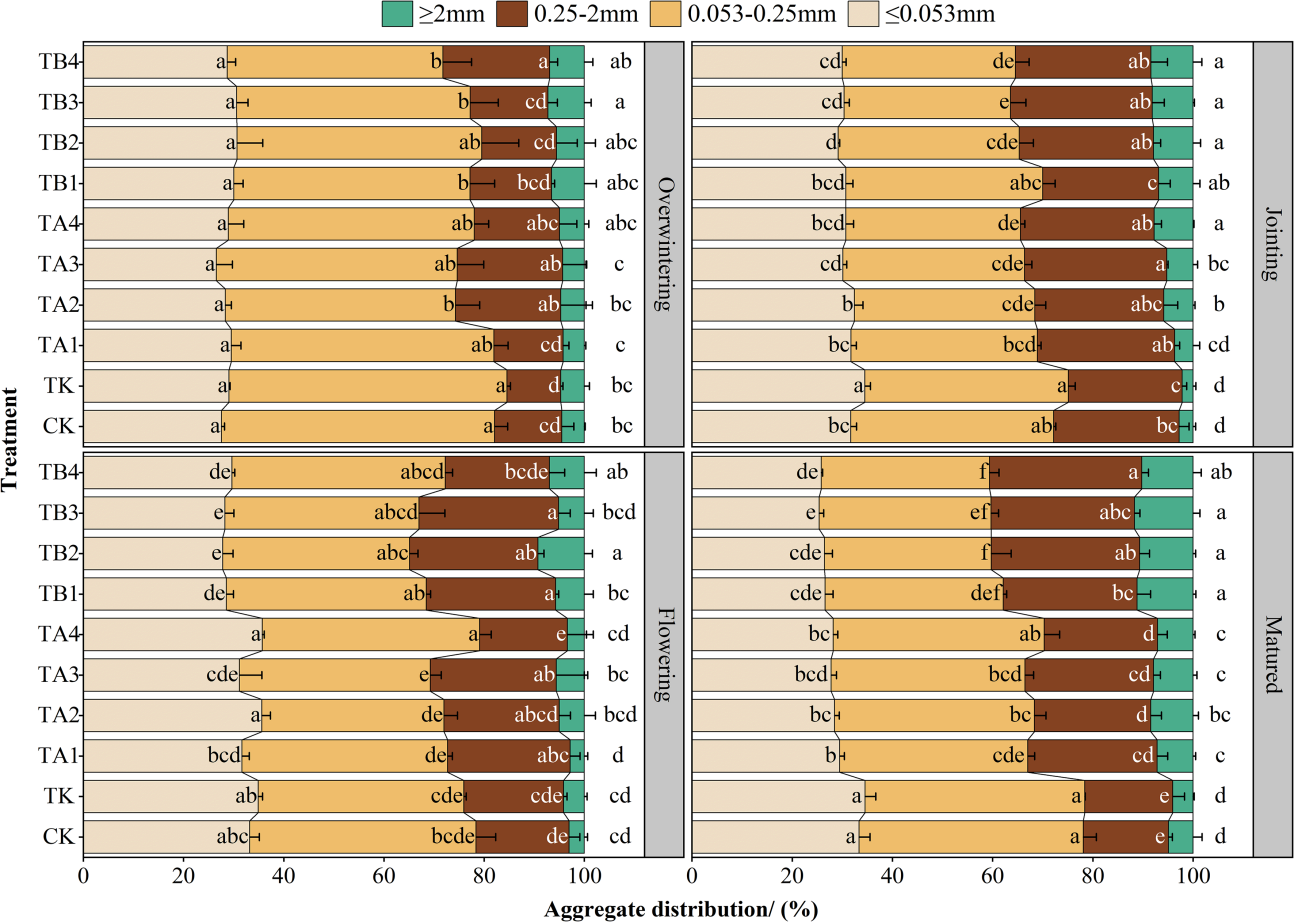
Effects of different fermentation methods of pig manure compost combined with chemical fertilizer on soil aggregate distribution of winter wheat. The different lowercase letters in the figure indicate significant differences between the treatments (*P* < 0.05), the same below.

The combined application of pig manure compost and chemical fertilizer promoted the transformation of <0.25 mm soil aggregates into >0.25 mm aggregates (**Figure 5**). In the CK and TK groups, the proportions of ≤0.053 mm and 0.053–0.25 mm aggregates increased over time, while the proportions of 0.25–2 mm and ≥2 mm aggregates decreased. In contrast, treatments TA1–TB4 showed the opposite trends: with the proportions of fine aggregates (≤0.053 and 0.053–0.25 mm) decreased, while those of the coarser aggregates (0.25–2 and ≥ 2 mm) increased with crop development. By maturity, TA1–TB4 treatments increased 0.25–2 mm and ≥2 mm aggregate proportions by an average of 8.73% and 6.42%, respectively, and reduced ≤0.053 mm and 0.053–0.25 mm aggregates by 6.78% and 4.52% compared to TK. Among treatments, the 50%:50% manure-to-fertilizer ratio group (TB1–TB4) showed the greatest improvement. Compared to TA1–TA4 treatments, TB1–TB4 treatments increased the proportion of 0.25–2 mm and ≥2 mm aggregates by 3.47% and 4.88%, respectively, and reduced the proportion of 0.053–0.25 mm and ≤0.053 mm aggregates by 2.79% and 3.96%, respectively.

### 3.7 Changes of soil microbial community

**Figure 6:**
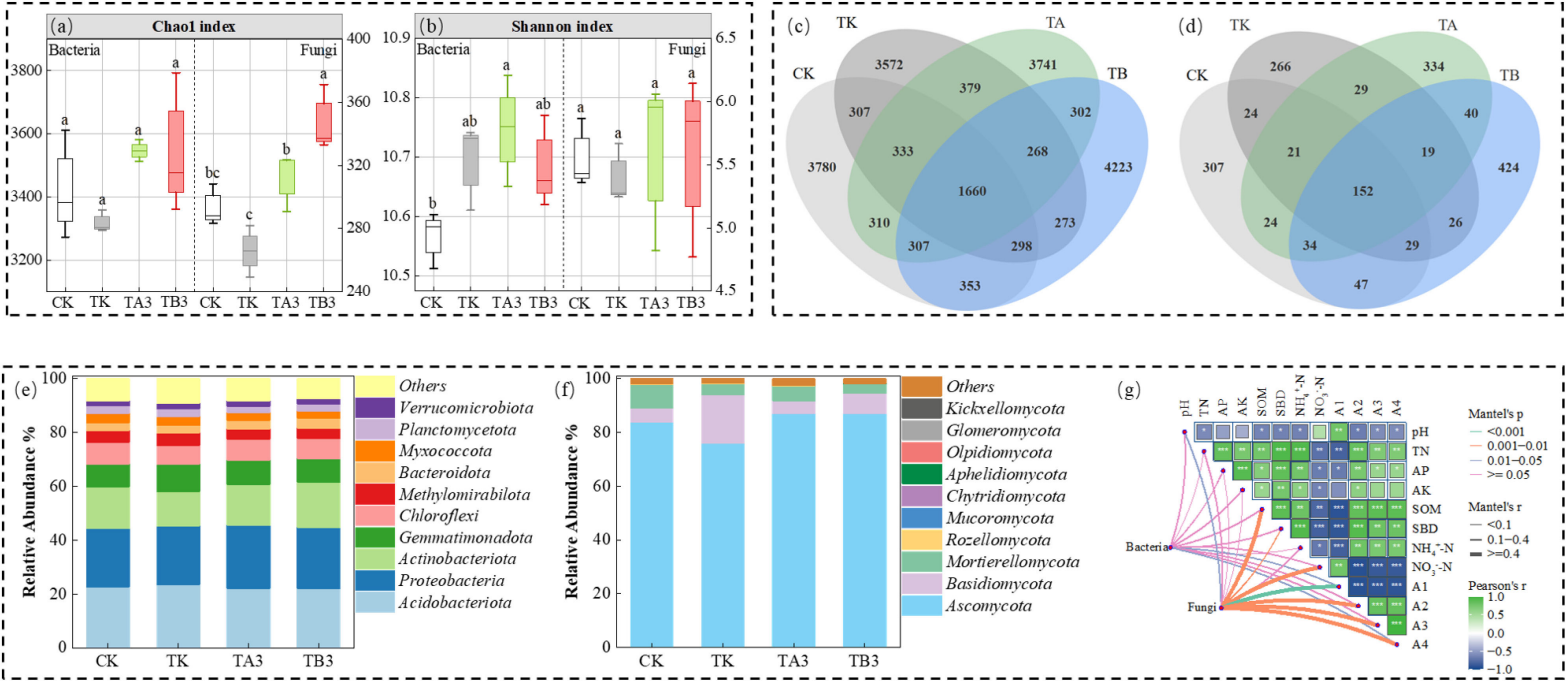
Effects of different fermentation methods of pig manure compost combined with chemical fertilizer on soil bacterial and fungal community composition of winter wheat. **(a)** Chao1 index of bacteria and fungi **(b)** Shannon index of bacteria and fungi, **(c)** Venn diagram of bacteria **(d)** Venn diagram of fungi, **(e)** relative abundance of the top 10 phyla of bacteria **(f)** relative abundance of the top 10 phyla of fungi, **(g)** heat map of soil physical and chemical indicators and Mantel test of bacterial and fungal diversity index. A4-large aggregates (≥2mm), A3-large aggregates (0.25-2mm), A2-microaggregates (0.053-0.25mm) and A1-powder clay (≤0.053mm).

Based on prior findings from our research group and the current analysis of plant and soil physicochemical indicators, the acid-regulated trough composting combined with chemical fertilizer application (TA3) was identified as the optimal organic-inorganic fertilization strategy (Wang et al., 2024b, Wang et al., 2025). To further investigate changes in functional microbial communities and their response mechanisms, soil microbial diversity was analyzed at maturity for CK, TK, TA3, and TB3 treatments. Compared to CK and TK, TA3 and TB3 significantly increased the Chao1 and Shannon indices for both bacterial and fungal communities (**Figures 6a, b**), indicating enhanced microbial diversity under organic–inorganic co-application. Venn diagram analysis (**Figures 6c, d**) showed that 1,660 bacterial OTUs and 152 fungal OTUs were shared across all treatments, suggesting that the core bacterial community was relatively stable, while the fungal community was more responsive to fertilization. Each treatment exhibited distinct OTUs, with TB3 showing the highest number of unique groups in both bacteria (4,223) and fungi (424), suggesting that a higher proportion of organic fertilizer substantially enhances microbial specificity and richness, thereby contributing to a more diverse soil microecosystem.

At the phylum level, a total of 33 bacterial phyla were detected across the four treatments (**Figure 6e**). The top ten phyla by relative abundance included *Acidobacteriota*, *Proteobacteria*, *Actinobacteriota*, *Gemmatimonadota*, *Chloroflexi*, *Methylomirabilota*, *Bacteroidota, Myxococcota, Planctomycetota, and Verrucomicrobiota*. Different treatments showed varying bacterial abundance. Compared to TK, the relative abundance of Acidobacteriota and Bacteroidota in TA3 and TB3 treatment groups increased by 0.17% and 1.07%, and 1.24% and 0.11%, respectively. For fungi (**Figure 6f**), 10 phyla were detected, with *Ascomycota, Basidiomycota, and Mortierellomycota* accounting for over 95% of the total abundance. *Ascomycota* dominated the fungal communities in TA3 and TB3, with relative abundances of 81.35% and 81.43%, slightly lower than those in TK by 1.35% and 1.27%. In contrast, *Basidiomycota* abundance increased by 0.26% and 2.40%, respectively.

Mantel test results (**Figure 6g**) indicated significant correlations between soil physicochemical properties, aggregate structure, and microbial community composition. Bacterial communities were strongly and positively correlated with nitrogen-related indicators (NH_4_^+^-N, NO_3_^-^-N, and total nitrogen), soil organic matter (SOM), available phosphorus (AP), and available potassium (AK) (Mantel’s *r* ≥ 0.4, *P* < 0.001). In addition, bacterial communities were significantly associated with large macroaggregates (A3, 0.25–2 mm) and very large aggregates (A4, ≥2 mm), suggesting that structurally stable, oxygen-rich macroaggregates provide a favorable microhabitat for bacteria. Although the correlations between fungal communities and environmental factors were slightly weaker, significant associations were still found with SOM, TN, AK, and medium-sized aggregates (A2, A3) (Mantel’s *r* > 0.3, *P* < 0.05), implying that fungi prefer well-aerated soils with moderate aggregate sizes and high organic matter. Notably, within the studied pH range, pH showed no significant correlation with either bacterial or fungal community structures. These findings were corroborated by heatmap and Pearson correlation analyses, further confirmed these findings, with bacterial communities showing stronger correlations with nitrogen, SOM, and AK compared to fungi, indicating that bacteria are more sensitive to nutrient fluctuations. In contrast, fungal communities responded more selectively to SOM and medium-sized aggregates.

**Figure 7:**
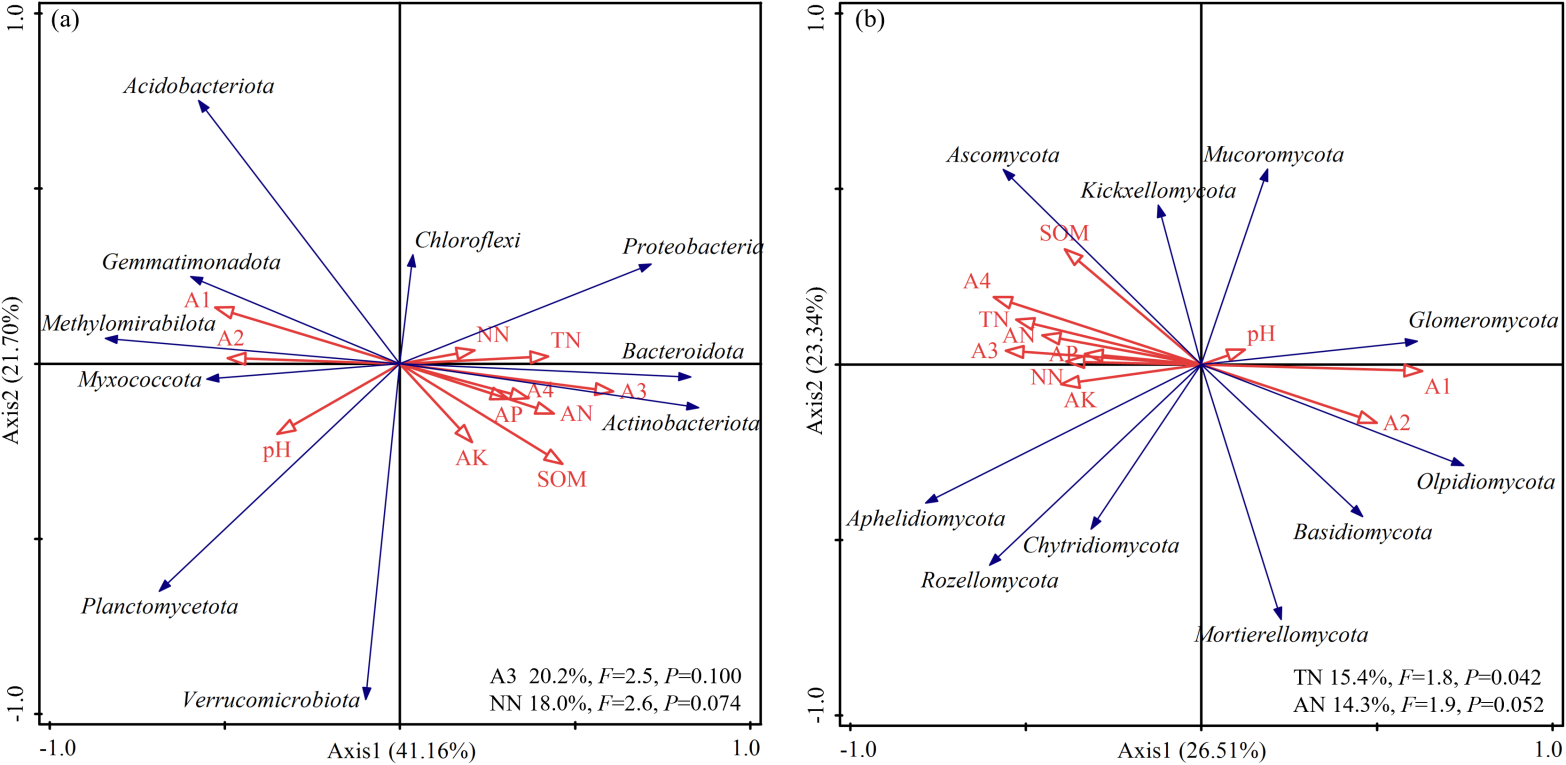
Redundancy analysis (RDA) of Bacteria **(a)** and Fungi **(b)** with Soil Properties. In the figure, AN-mmonium nitrogen, NN-nitrate nitrogen, A4-large aggregates (≥2mm), A3-large aggregates (0.25-2mm), A2-microaggregates (0.053-0.25mm) and A1-powder clay (≤0.053mm).

**Figure 7a** illustrates the relationship between bacterial phylum-level community structure and various environmental factors, with RDA1 and RDA2 explaining 39.48% and 27.13% of the variation, respectively. *Proteobacteria*, *Bacteroidota*, and *Actinobacteriota* were significantly positively correlated with environmental factors, including TN, NO_3_^-^-N, NH_4_^+^-N, AP, AK, and SOM. In contrast*, Acidobacteriota, Gemmatimonadota*, and *Methylomirabilota* were distributed in the opposite directions, suggesting their higher abundance under oligotrophic or stable soil conditions. *Chloroflexi*, *Planctomycetota*, and *Verrucomicrobiota* appeared toward the central-left region of the plot, indicating weak correlations with the environmental variables, possibly reflecting low sensitivity to environmental changes or broad ecological tolerance. **Figure 7b** shows the relationship between fungal phylum-level communities and soil environmental factors, with RDA1 and RDA2 explaining 22.65% and 19.38% of the variation, respectively. *Ascomycota*, *Mucoromycota*, and *Kickxellomycota* were significantly positively associated with nutrient-rich conditions, including SOM, NH_4_^+^-N, TN, NO_3_^-^-N, and AP, suggesting their greater activity or dominance in fertile soils. Conversely, *Glomeromycota*, *Olpidiomycota*, and *Mortierellomycota* were distributed along gradients of higher pH and lower nutrient levels, indicating a preference for neutral to alkaline, nutrient-poor environments. *Basidiomycota*, *Chytridiomycota*, and *Rozellomycota* were positioned in the upper-left quadrant, implying weak responses to the main environmental variables assessed. Among the factors, TN (*F* = 1.8, *P* = 0.04) and NH_4_^+^-N (*F* = 1.9, *P* = 0.05) significantly influenced fungal community composition, explaining 15.4% and 14.3% of total variance, respectively.

**Figure 8:**
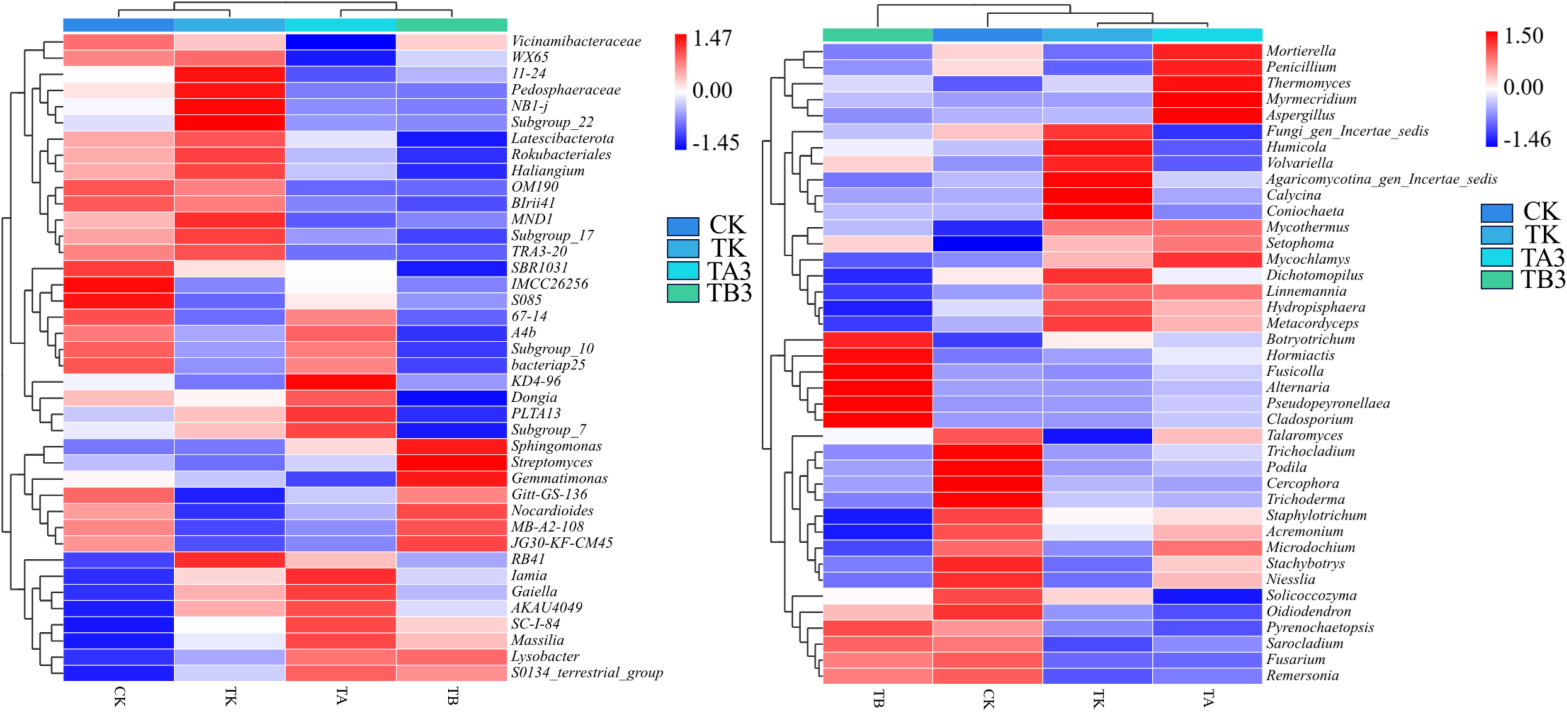
Effects of pig manure compost with different fermentation methods combined with chemical fertilizer on the species composition heat map of bacteria (left) and fungi (right) in winter wheat soil.

The species composition heatmap (**Figure 8**) further illustrated the impact of different fertilization treatments on soil bacterial and fungal community structures. In the bacterial community, compared with CK, TK treatment significantly enriched taxa such as *Vicinamibacteraceae*, *WQ55*, *Il-24*, and *NB1-j*, indicating their sensitivity to readily available inorganic nutrients. In contrast, genera like *Massilia* and *S0154_terrestrial_group* were more abundant under CK, suggesting a preference for low-nutrient environments. With increasing proportions of organic fertilizer, compost-related taxa such as *Streptomyces*, *Gaiella*, and *Gemmatimonas* became progressively enriched in TA3 and TB3, with TB3 showing the most pronounced accumulation, reflecting their affinity for compost-derived carbon sources and organic matter. In the fungal community, the abundances of *Aspergillus*, *Trichoderma*, and *Talaromyces* decreased under TK but significantly rebounded in TB3, indicating that organic matter input supports their growth. Saprophytic fungi such as *rtierella*, *Penicillium*, and *Humicola* were notably enriched in TB3, suggesting that higher compost ratios promote functional fungal proliferation and nutrient cycling. Conversely, *Stachybotrys* and *Sarocladium* were more abundant in CK, indicating their adaptation to nutrient-poor or undisturbed environments. Overall, among the organic-inorganic combined treatments, TB3 had the most pronounced effect, contributing to enhanced microbial diversity and improved ecological function in the soil.

### 3.8 Soil properties, interaction between microorganisms and plants after application of pig manure compost

**Figure 9:**
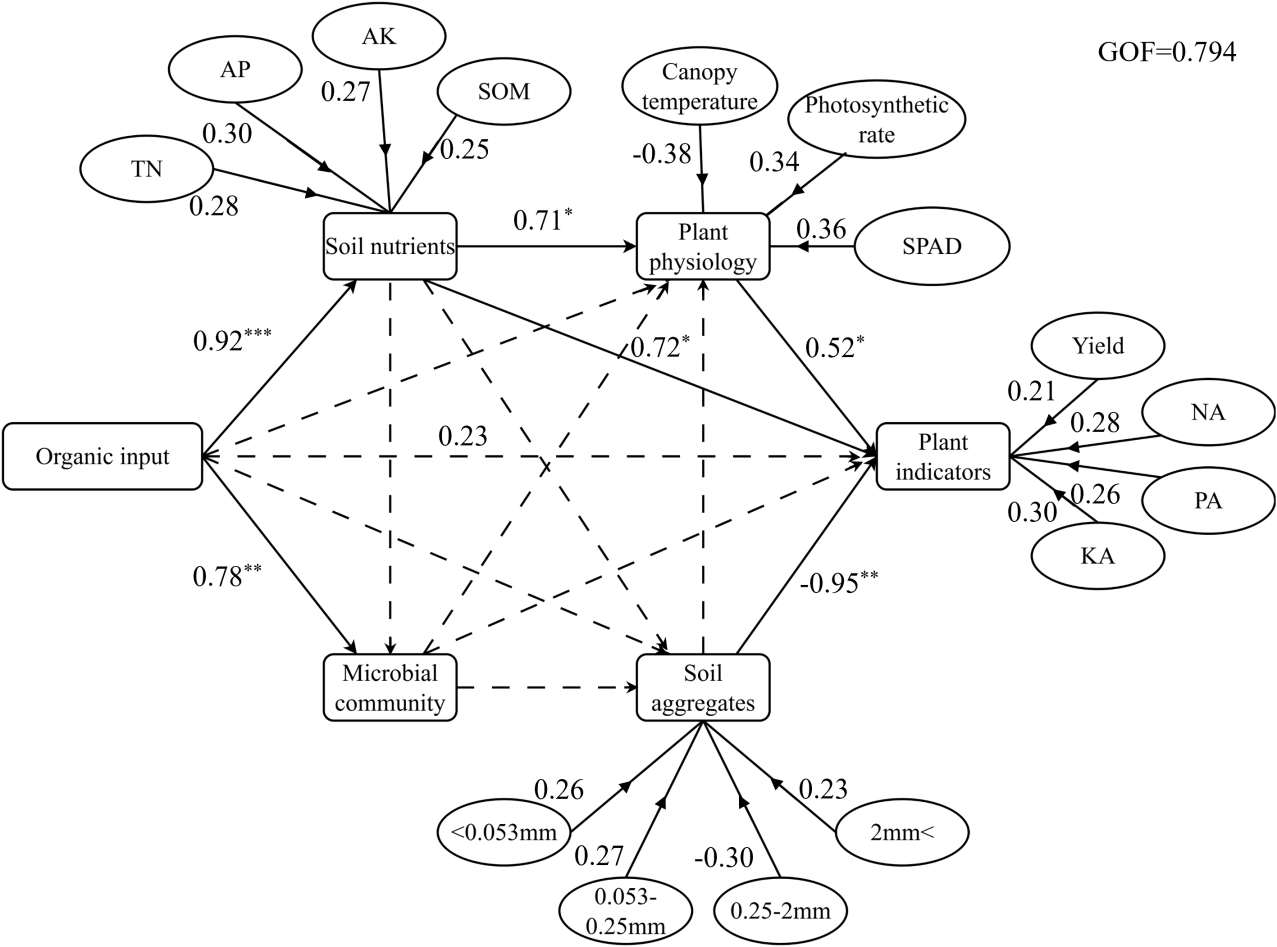
Partial least squares path analysis model. The TN, AP, AK and SOM is soil total nitrogen, available phosphorus, available potassium and organic matter. < 0.053mm, 0.053-0.25mm, 0.25-2mm and 2mm < is aggregate size distribution. NA, PA and KA is the accumulation of nitrogen, phosphorus and potassium.

The partial least squares path model was used to evaluate the effects of fertilization and soil factors on yield (**Figure 9**). The results showed that pig manure composting had a significant positive effect on soil nutrients (Path coefficient = 0.92, *P* < 0.001) and microbial communities (Path coefficient = 0.78, *P* < 0.01). In addition, soil nutrients significantly positively affected plant indicators (Path coefficient = 1.52, *P* < 0.001) and plant physiology (Path coefficient = 0.71, *P* < 0.01), and soil aggregates (Path coefficient = -0.95, *P* < 0.01) significantly negatively affected plant indicators. The indirect effect of pig manure compost on plant indicators was greater than its direct effect (Path coefficient = 0.23), indicating that the application of pig manure compost may indirectly affect crop yield and nutrient accumulation by changing soil nutrient levels or improving plant physiological status.

## 4 Discussion

### 4.1 Yield, nutrients, light and temperature physiology

Integrated application of organic and inorganic fertilizers is an effective strategy to improve crop yield and to enhance N, P, and K accumulation (Gelaye, 2023; Hu et al., 2023; Li et al., 2022a; He et al., 2022). Organic fertilizers, rich in organic matter and diverse nutrients, help maintain an optimal C: N ratio and provide sustained nutrient availability. Their application with chemical fertilizers improves nutrient retention and supply, promotes nutrient uptake, and enhances N, P, and K accumulation—ultimately boosting fertilizer use efficiency and crop yield (Verma et al., 2023; Wei et al., 2020; Gorfie et al., 2022; He et al., 2023). In this study, treatments TA1–TA4 (25% organic + 75% chemical fertilizer) significantly increased wheat yield, thousand-grain weight, and N, P, and K accumulation compared with TK, whereas treatments TB1–TB4 (50% organic + 50% chemical fertilizer) showed a declining trend for the same indicators (**Table. 2**). This may be attributed to insufficient levels of available nutrients in higher organic fertilizer treatments during peak wheat nutrient demand, resulting in poor nutrient synchrony. High organic input also disrupts the soil C: N balance, stimulating heterotrophic microbial activity. These microbes preferentially utilize mineral nitrogen for their metabolism and organic matter decomposition, causing “nitrogen immobilization,” which suppresses plant growth and nutrient accumulation (Mooshammer et al., 2014). Logistic modeling results (**Figure 2**, **Supplementary Table S1**) indicated that 25% organic fertilizer substitution (TA treatments) enhanced nutrient accumulation rates and accelerated winter recovery, advancing wheat growth resumption. However, lower availability of mineral nutrients in TB treatments restricted nutrient uptake compared to TA and TK. This is likely due to organic fertilizers increasing soil water content and specific heat, buffering against temperature fluctuations, and protecting roots and tillers from frost. They also enhance the activities of β-xylosidase and α-glucosidase, promoting straw decomposition and accumulation of labile carbon (e.g., EOC, DOC), supplying energy during greening (Gong et al., 2019). Moreover, a 25-year long-term fertility trial found no yield advantage from combined fertilization over single applications of equivalent nutrient levels from either organic or inorganic sources. This outcome highlights the complex interactions among fertilizers, cropping systems, soil properties, and field management, and underscores the need to tailor fertilization strategies to local soil fertility, environmental conditions, and agronomic practices (Ripoche et al., 2015). The difference chiefly reflects soil and management factors: the Mali experiment was on low-fertility sandy soils with weak nutrient buffering and manure inputs already large relative to mineral fertilizer in a cotton–sorghum–groundnut rotation, whereas this study involved higher-fertility sandy fluvo-aquic soils and optimized nutrient ratios and timing for winter wheat, so organic–mineral co-application produced a clear yield benefit here.

Maintaining an optimal light-temperature physiological state is essential for achieving high crop yields. Canopy temperature, reflecting the energy exchange between plant leaves and their environment, serves as an indicator of leaf water status and photosynthetic activity (Mao et al., 2025). It is closely linked to photosynthesis as elevated canopy temperature often signal water deficits or environmental stress, leading to stomatal closure, reduced stomatal conductance, and suppressed transpiration, which collectively decrease CO_2_ uptake and significantly reduce the photosynthetic rate (Jiang et al., 2024; Hill et al., 2014). In this study, organic fertilizers produced through different fermentation methods reduced wheat canopy temperature by an average of 4.55% and enhanced leaf Pn (**Supplementary Table S2**; **Supplementary Figure S1**), consistent with findings by Jiang et al. (2024) and Xiao et al. (2022a). High temperature negatively impacts chlorophyll stability and enzymatic activity in leaves, thereby lowering photosynthetic efficiency. In contrast, reduced canopy temperature is associated with higher leaf water content and physiological activity, greater stomatal opening, enhanced transpiration, and improved gas exchange. This allows more CO_2_ to be absorbed for photosynthesis, ultimately increasing photosynthetic rate and productivity. Thus, monitoring canopy temperature provides a rapid assessment of photosynthetic performance and overall plant health. Maintaining an optimal canopy temperature is not only a sign of healthy wheat development but also a fundamental physiological basis for efficient photosynthesis and high yield. Pig manure compost improves soil water retention and nutrient-holding capacity, enhancing root water and nutrient uptake, which alleviates water stress and helps keep stomata open, thereby lowering canopy temperature. Additionally, the slow and steady nutrient release from compost ensures a more stable nitrogen supply, which increases chlorophyll content and delays leaf senescence. This contributes to a higher leaf area index and sustained stomatal conductance, improving CO_2_ assimilation and fixation capacity, and ultimately boosting photosynthetic rate (Xiao et al., 2022b).

### 4.2 Soil nutrients and physical structure

Plant growth and development are strongly influenced by soil physical structure and chemical properties. Sandy fluvo-aquic soils, while well-aerated due to their loose texture, often suffer from poor water retention, leading to drought stress, nutrient leaching, and restricted root development, all of which limit crop growth and yield formation (Zhang et al., 2021a). Studies have shown that the combined application of organic and inorganic fertilizers improves soil nutrient content, enhances aggregate structure, and increases aggregate stability (Lu et al., 2024; Ren et al., 2025). In this study, pig manure compost combined with chemical fertilizers increased levels of total N, ammonium-N, nitrate-N, available P, K, and soil organic matter (**Supplementary Figure S2**), while also increasing the proportion of soil aggregates ≥0.25 mm(**Figure 5**), consistent with findings by Guo et al. (2022) and Zhang et al. (2016). During the overwintering stage, the TK treatment had higher initial nutrient levels due to its readily available nutrients. However, its weak retention capacity led to a decline in nutrient availability from green-up to heading, making it difficult to sustain wheat nutrient demands. In contrast, treatments with pig manure compost provided a stable nutrient supply throughout the growing season, particularly during the jointing stage when nutrient demand peaks and available nutrient levels in TK decline. Oorts et al. (2007) found a significant positive correlation between aggregate content/stability and soil fertility. Organic matter in compost improves colloidal capacity for nutrient adsorption, reducing nutrient loss risks. Moreover, compost enhances microbial secretion of binding agents and fungal hyphal networks, promoting the formation and stabilization of macroaggregates (Wang et al., 2024a; Tian et al., 2025). In contrast, sole application of chemical fertilizers can accelerate microbial mineralization of organic matter, leading to soil structure collapse and reduced aggregate stability (Zhang et al., 2021b). Long-term use of chemical fertilizers in paddy soils has also been linked to structural degradation, whereas organic amendments significantly enhance soil aggregate stability (Guo et al., 2019).

Both NH_4_^+^-N and NO_3_^-^-N are plant-available nitrogen forms, but due to their mobility, they often leach into groundwater through irrigation or rainfall. Our results showed that NH_4_^+^-N and NO_3_^-^-N were mainly concentrated in the 0–20 cm and 20–100 cm soil layers, respectively. Compared to chemical fertilizers alone, combined pig manure treatments significantly reduced NH_4_^+^-N and NO_3_^-^-N levels in the 20–100 cm soil layer (**Figure 4**), aligning with Abbasi and Sepaskhah (2023) and Li et al. (2024b). Surface-applied nitrogen creates inorganic nitrogen-rich microzones that convert to NO_3_^-^-N via nitrification. Precipitation events (e.g., snow and rain) during the growth period can then drive NO_3_^-^-N downward. Hu et al. (2022) noted that chemical fertilizers increase nitrifying gene abundance, accelerating NH_4_^+^-N to NO_3_^-^-N conversion, while compost fosters denitrification. Quan et al. (2016) reported that organic fertilizers can inhibit nitrification, as a high C: N ratio encourages NH_4_^+^-N immobilization, preventing NO_3_^-^-N leaching. Furthermore, compost modifies soil surface charge, stimulating microbial nitrogen cycling processes that enhance NO_3_^-^-N adsorption, immobilization, and assimilation, reducing vertical nitrogen loss (Wen et al., 2024; Asiloglu et al., 2021). In early wheat growth stage, nitrogen demand is low. Nitrogen from compost is adsorbed and retained in the upper soil layers, limiting downward migration. Since nitrogen leaching is mainly driven by vertical water movement, and the tested sandy soil is prone to nutrient loss, compost application promotes microbial synthesis of binding agents, reinforcing inter-particle adhesion and hydrophobicity. This promotes the formation of larger aggregates, optimizes the soil’s three-phase structure, increases porosity, and enhances water retention. Collectively, these effects limit NO_3_^-^-N transport through the soil profile, maintain inorganic nitrogen in upper layers, and prevent groundwater contamination (Tian et al., 2022).

### 4.3 Soil enzyme activity and microbial community composition

Soil enzyme activity is a critical biological indicator of soil quality, fertility, and microecological function. These enzymes directly participate in biogeochemical cycling and nutrient transformation and are widely used to assess soil biochemical functionality and ecosystem health (Jabborova et al., 2021). Integrating organic and inorganic fertilizers is considered an effective strategy for enhancing enzyme activity and unlocking soil productivity (Zhang et al., 2022; He et al., 2020). This study shows that pig manure compost significantly increased soil enzyme activities (S-URE, S-ACP, S-DHA), with the TB treatment exhibiting the greatest enhancement (**Table 3**). This improvement is primarily attributed to the high organic carbon content in the compost, which provides a stable carbon source that promotes microbial growth and enzyme secretion. Additionally, the compost is rich in soluble organics and microbial metabolites that help create a favorable microecological environment, enhancing enzyme system stability and activity, thereby improving nutrient transformation and soil fertility—an essential foundation for sustainable crop productivity (Jabborova et al., 2021). Soil microorganisms, as key drivers of nutrient cycling, soil structure, and plant growth, profoundly influence agroecosystem stability and productivity (Kennedy and Smith, 1995). Studies have shown that organic fertilization can reshape microbial community structures and increase the abundance of beneficial taxa (Li et al., 2022b; Sun et al., 2024). In this study, TA3 and TB3 treatments increased bacterial and fungal α-diversity and OTU richness (**Figures 6a-d**), suggesting that compost not only maintains core microbial groups but also promotes the colonization and expansion of rare taxa by providing diverse carbon sources and energy substrates. This diversifies microbial niches, enhances functional redundancy, and stabilizes the community (Hartmann et al., 2015; Chen et al., 2020). However, the limited increase in Chao1 and Shannon indices may be due to the relatively short duration of organic input.

At the phylum level, the relative abundances of *Proteobacteria, Actinobacteriota, Bacteroidota*, and *Ascomycota* were significantly higher for TA3 and TB3 than other treatments (**Figures 6e-f**). These groups can decompose organic matter and mineralize nutrients, enhancing enzyme activities such as S-URE, S-ACP, and S-DHA, accelerating nitrogen and phosphorus cycling (Zhou et al., 2016; Lazcano et al., 2013). Compost application provided abundant carbon sources for heterotrophic microorganisms, promoting their growth and community diversity, whereas fertilizer alone was rapidly utilized by a few fast-acting microorganisms, reducing overall diversity. RDA results further indicate that microbial community changes are primarily driven by factors such as SOM, pH, AP, and NH_4_^+^-N. These environmental factors influence microbial metabolism by regulating nutrient availability and microhabitat conditions. Consequently, microbially mediated nutrient release enhances wheat nutrient accumulation, photosynthetic activity, and yield. Among treatments, the acid-regulated compost demonstrated optimal performance. This may result from its ability to promote the enrichment of beneficial microorganisms—such as actinomycetes, nitrogen-fixing bacteria, and phosphorus-solubilizing bacteria—by regulating pH during fermentation. This process enhances enzyme activity and accelerates humus formation. Upon soil incorporation, organic acids further improve the rhizosphere environment, enhance root nutrient uptake, and stabilize nutrient supply systems, thereby increasing nutrient use efficiency and yield.

By comparing natural composting, water-regulated trough composting, acid-regulated trough composting, and conventional trough composting, significant differences in nutrient supply, soil structure, enzyme activity, and microbial communities were showed among the four fermentation methods. Owing to the lack of environmental regulation and decomposes slowly, natural composting leads to a significant nutrient loss. Conventional trough composting improves decomposition conditions through aeration, but moisture and pH fluctuations still limit nutrient retention and microbial activity. Water-regulated trough composting, by stabilizing moisture content, promotes organic matter degradation and humus formation, thereby enhancing the sustained nutrient supply and soil enzyme activity (Zhou et al., 2022). In contrast, acid-regulated trough composting exhibits the best overall performance: organic acids could regulate pH, reduce nitrogen volatilization (Pan et al., 2018; Nie et al., 2020), enrich beneficial communities (Li et al., 2022), enhance urease and acid phosphatase activity, and accelerate the mineralization and transformation of nutrients (Miao et al., 2024). Furthermore, acid regulation promotes humus production and reduces the bioavailability of heavy metals (Wu et al., 2021; Li et al., 2022), significantly enhance the compost’s water and nutrient retention and slow-release properties (Cao et al., 2019). In summary, acid-regulated trough composting could significantly improve the quality and fertilizer efficiency of compost, improve the soil environment and promote crop growth, and thus is the optimal fermentation method for treating pig manure.

## 5 Conclusions

Compared with chemical fertilizer alone, the application of acetic acid-regulated pig manure compost combined with chemical fertilizer effectively reconstructed soil aggregate composition by increasing the proportion of ≥0.25 mm aggregates and reducing nitrogen leaching losses. It also enhanced soil enzyme activities (S-URE, S-ACP, S-DHA), improved microbial diversity, and boosted nutrient-supplying capacity. These improvements promoted nutrient uptake and accumulation in winter wheat roots, enhanced canopy photosynthetic performance, and ultimately increased yield. Among the four fermentation methods, the acid-regulated trough compost exhibited the most balanced and superior effects, as it not only minimized nitrogen loss and enhanced enzymatic activities but also promoted beneficial microbial proliferation and humus formation. Therefore, the acetic acid-regulated trough-compost combined with chemical fertilizer at a 25:75 ratio demonstrated the best overall performance, making it a promising strategy for sustainable winter wheat cultivation on sandy fluvo-aquic soils in northern Henan.

## Data Availability

The raw data supporting the conclusions of this article will be made available by the authors, without undue reservation.
