# Supplementary material for: Effects of combined application of pig manure composts prepared using different fermentation methods with chemical fertilizer on winter wheat yield, light-thermal physiology, and soil biological characteristics
Source: Front Plant Sci. 2025 Oct 31;16:1711898. doi: 10.3389/fpls.2025.1711898 (PMC12615480; doi:10.3389/fpls.2025.1711898)
Supplement: Supplementary file 1 [file DataSheet1.docx]

**Effects of Combined Application of Pig Manure Composts Prepared Using Different Fermentation Methods with Chemical Fertilizer on Winter Wheat Yield, Light-thermal Physiology, and Soil Biological Characteristics**

Mingteng Wang ^a, 1^, Jiaming Cai ^a, 1^, Chong Zeng ^a^, Meng Li ^a^, Yilun Wang ^a^, Sainan Geng ^a^, Gang Li ^b^, Lantao Li ^a, *^

^a^ College of Resources and Environment, Henan Agricultural University, Zhengzhou 450046, China

^b^ College of Mechanical & Electrical Engineering, Henan Agricultural University, Zhengzhou 450046, China

**^1^ The co-authors contributed equally to this work.**

*** Corresponding author** Lantao Li, Mobile: +86-15136260916, E-mail: lilantao@henau.edu.cn

**Address:** College of Resources and Environment, Henan Agricultural University, No.218 Ping'an Avenue, Zhengdong New District, Zhengzhou, 450046, PR China.

**Tel:** +86 (0371) 63555504

**Fax:** +86 (0371) 63555504

**E**-**mail:** [lilantao@henau.edu.cn](mailto:lilantao@henau.edu.cn)

**Table S1** Effects of different fermentation methods of pig manure compost combined with chemical fertilizer on nutrient accumulation dynamic model and its parameters of winter wheat

| Element | Treatment | Regression equations | Y_m_  (kg·hm^-2^) | t_1_  (d) | t_2_  (d) | Δt  (d) | T_max_  (d) | V_max_  (kg·hm^-2^·d^-1^) | V_mean_  (kg·hm^-2^·d^-1^) | *R*^2^ |
| --- | --- | --- | --- | --- | --- | --- | --- | --- | --- | --- |
| N | CK | y=117.25/(1+e^(5.3910-0.0344x)^) | 122.46 | 114.18 | 202.79 | 88.61 | 158.48 | 0.91 | 0.61 | 0.9950 |
|  | TK | y=168.10/(1+e^(5.1995-0.0340x)^) | 168.10 | 114.35 | 191.93 | 77.58 | 153.14 | 1.43 | 0.95 | 0.9911 |
|  | TA1 | y=169.45/(1+e^(5.5250-0.0371x)^) | 169.49 | 113.35 | 184.31 | 70.95 | 148.83 | 1.57 | 1.05 | 0.9969 |
|  | TA2 | y=174.66/(1+e^(5.5574-0.0376x)^) | 174.66 | 112.85 | 182.95 | 70.10 | 147.90 | 1.64 | 1.09 | 0.9957 |
|  | TA3 | y=173.98/(1+e^(5.5818-0.0399x)^) | 173.98 | 106.76 | 172.70 | 65.94 | 139.73 | 1.74 | 1.16 | 0.9982 |
|  | TA4 | y=165.21/(1+e^(5.2922-0.0359x)^) | 165.21 | 110.83 | 184.26 | 73.43 | 147.54 | 1.48 | 0.99 | 0.9976 |
|  | TB1 | y=147.22/(1+e^(4.5956-0.0310x)^) | 147.22 | 105.84 | 190.86 | 85.03 | 148.35 | 1.14 | 0.76 | 0.9986 |
|  | TB2 | y=144.87/(1+e^(4.5767-0.0315x)^) | 144.87 | 103.54 | 187.20 | 83.66 | 145.37 | 1.14 | 0.76 | 0.9959 |
|  | TB3 | y=154.26/(1+e^(4.7173-0.0313x)^) | 154.26 | 108.47 | 192.49 | 84.02 | 150.48 | 1.21 | 0.81 | 0.9995 |
|  | TB4 | y=146.11/(1+e^(4.7186-0.0314x)^) | 146.12 | 108.43 | 192.39 | 83.96 | 150.41 | 1.15 | 0.76 | 0.9997 |
| P | CK | y=19.96/(1+e^(5.5446-0.0367x)^) | 19.96 | 115.16 | 186.91 | 71.75 | 151.03 | 0.18 | 0.12 | 0.9978 |
|  | TK | y=32.51/(1+e^(5.6777-0.0382x)^) | 32.51 | 114.28 | 183.30 | 69.03 | 148.79 | 0.31 | 0.21 | 0.9897 |
|  | TA1 | y=34.52/(1+e^(5.8701-0.0418x)^) | 34.52 | 108.84 | 171.81 | 62.97 | 140.33 | 0.36 | 0.24 | 0.9995 |
|  | TA2 | y=33.82(1+e^(5.8464-0.0423x)^) | 33.82 | 106.88 | 169.03 | 62.15 | 137.95 | 0.36 | 0.24 | 0.9989 |
|  | TA3 | y=35.38/(1+e^(5.8786-0.0440x)^) | 35.38 | 103.73 | 163.63 | 59.90 | 133.68 | 0.39 | 0.26 | 1.0000 |
|  | TA4 | y=32.88/(1+e^(5.7329-0.0409x)^) | 32.88 | 108.04 | 172.48 | 64.44 | 140.26 | 0.34 | 0.22 | 0.9990 |
|  | TB1 | y=26.87/(1+e^(5.3563-0.0379x)^) | 26.87 | 106.71 | 176.30 | 69.58 | 141.50 | 0.25 | 0.17 | 0.9993 |
|  | TB2 | y=26.11/(1+e^(5.5899-0.0403x)^) | 26.11 | 106.04 | 171.41 | 65.37 | 138.73 | 0.26 | 0.18 | 0.9989 |
|  | TB3 | y=28.27/(1+e^(5.5968-0.0390x)^) | 28.27 | 109.88 | 177.50 | 67.62 | 143.69 | 0.28 | 0.18 | 0.9973 |
|  | TB4 | y=26.35/(1+e^(5.5627-0.0388x)^) | 26.35 | 109.35 | 177.18 | 67.84 | 143.27 | 0.26 | 0.17 | 0.9961 |
| K | CK | y=126.05/(1+e^(5.7022-0.0360x)^) | 126.05 | 104.33 | 177.52 | 73.18 | 140.93 | 1.13 | 0.76 | 0.9971 |
|  | TK | y=166.00/(1+e^(6.2830-0.0404x)^) | 166.00 | 122.97 | 188.20 | 65.23 | 155.59 | 1.68 | 1.12 | 0.9896 |
|  | TA1 | y=165.11/(1+e^(6.5662-0.0434x)^) | 165.11 | 120.83 | 181.46 | 60.63 | 151.15 | 1.79 | 1.20 | 0.9963 |
|  | TA2 | y=169.86/(1+e^(6.5150-0.0433x)^) | 169.86 | 120.01 | 180.82 | 60.81 | 150.41 | 1.84 | 1.23 | 0.9977 |
|  | TA3 | y=173.60/(1+e^(6.5467-0.0443x)^) | 173.60 | 118.02 | 177.46 | 59.44 | 147.74 | 1.92 | 1.28 | 0.9974 |
|  | TA4 | y=164.98/(1+e^(6.4972-0.0427x)^) | 164.98 | 121.47 | 183.24 | 61.77 | 152.36 | 1.76 | 1.17 | 0.9954 |
|  | TB1 | y=151.65/(1+e^(6.0845-0.0399x)^) | 151.65 | 119.43 | 185.42 | 65.99 | 152.42 | 1.51 | 1.01 | 0.9988 |
|  | TB2 | y=152.00/(1+e^(6.0457-0.0401x)^) | 152.00 | 117.87 | 183.53 | 65.66 | 150.70 | 1.52 | 1.02 | 0.9989 |
|  | TB3 | y=156.26/(1+e^(6.0748-0.0398x)^) | 156.26 | 119.40 | 185.51 | 66.10 | 152.46 | 1.56 | 1.04 | 0.9979 |
|  | TB4 | y=156.62/(1+e^(6.0263-0.0383x)^) | 156.62 | 122.94 | 191.70 | 68.76 | 157.32 | 1.50 | 1.00 | 0.9987 |

In the table, y is the nutrient accumulation of winter wheat, and x is the days after sowing; Y_m_ is the maximum nutrient accumulation, V_max_ is the maximum accumulation rate, V_mean_ is the average accumulation rate, t_1_ is the start date of rapid accumulation, t_2_ is the end period of rapid accumulation, Δt is the duration of rapid nutrient accumulation in winter wheat, and R^2^ is the fitting correlation coefficient.

**Table S2** Effects of different fermentation methods of pig manure compost and chemical fertilizer on canopy temperature of winter wheat

| Treatment | Overwintering (°C) | | | |  | | Jionting (°C) | | |  | Floweing (°C) | | |  |
| --- | --- | --- | --- | --- | --- | --- | --- | --- | --- | --- | --- | --- | --- | --- |
|  | Minimum temperature | Maximum temperature | Average temperature |  | | Minimum temperature | | Maximum temperature | Average temperature |  | Minimum temperature | Maximum temperature | Average temperature | |
| CK | 6.40±0.95a | 9.40±0.50a | 7.60±0.95a |  | | 20.50±0.36 a | | 24.93±0.60 a | 22.23±0.25 a |  | 22.73±0.50 a | 26.90±0.89 a | 25.47±0.49 a | |
| TK | 6.03±0.32a | 9.30±0.96a | 7.63±0.31a |  | | 20.40±0.26 ab | | 24.40±0.00 ab | 22.07±0.21 ab |  | 22.33±0.40 a | 26.50±1.71 a | 24.67±0.25 ab | |
| TA1 | 5.73±0.21a | 8.47±0.12abc | 7.20±0.30a |  | | 20.20±0.44 abc | | 24.10±1.04 ab | 21.57±0.42 bc |  | 21.93±0.90 a | 26.30±0.60 a | 23.60±0.62 bc | |
| TA2 | 5.80±0.21a | 8.50±0.56abc | 7.10±0.20a |  | | 19.87±0.21 bc | | 24.07±0.25 ab | 21.50±0.30 c |  | 21.40±1.11 a | 25.80±0.98 a | 23.43±0.86 bc | |
| TA3 | 5.73±1.07a | 8.23±0.87c | 6.93±1.11a |  | | 19.70±0.56 c | | 23.50±0.44 b | 21.27±0.32 c |  | 21.27±0.49 a | 25.50±0.20 a | 22.83±0.59 c | |
| TA4 | 5.80±0.66a | 8.63±0.47abc | 7.07±0.42a |  | | 19.87±0.12 bc | | 23.77±0.25 b | 21.30±0.26 c |  | 21.93±1.16 a | 25.90±0.78 a | 24.17±0.40 abc | |
| TB1 | 5.90±0.26a | 9.30±0.20a | 7.43±0.21a |  | | 20.10±0.26 abc | | 24.07±0.38 ab | 21.67±0.12 bc |  | 22.00±0.85 a | 26.43±0.76 a | 23.97±0.75 bc | |
| TB2 | 5.88±0.32a | 9.23±0.38ab | 7.07±0.15a |  | | 20.33±0.06 ab | | 24.13±0.45 ab | 21.73±0.29 bc |  | 22.03±0.35 a | 25.80±1.01 a | 23.63±1.23 bc | |
| TB3 | 5.85±0.50a | 9.07±0.29abc | 6.97±1.14a |  | | 19.93±0.49 bc | | 24.00±1.23 ab | 21.63±0.25 bc |  | 22.00±0.70 a | 25.60±0.98 a | 23.50±0.53 bc | |
| TB4 | 5.90±1.15a | 8.30±0.40bc | 6.97±0.38a |  | | 20.03±0.46 abc | | 24.17±0.51 ab | 21.73±0.06 bc |  | 21.97±1.36 a | 25.73±1.00 a | 23.83±1.19 bc | |

Different lowercase letters in the same row indicate significant differences between treatments (*P*<0.05).

**Fig. S1**

Effect of different fermentation methods of pig manure compost with chemical fertilizer on photosynthetic parameters of winter wheat. The different lowercase letters in the figure indicate significant differences between the treatments (P<0.05), the same below.

**Fig. S2**

Effect of different fermentation methods of pig manure compost with chemical fertilizer on the nutrient content of winter wheat soils
